# Benefits of uric acid-lowering medication after bariatric surgery in patients with gout

**DOI:** 10.1186/s12893-024-02472-6

**Published:** 2024-06-14

**Authors:** Ke Song, Ming He, Xiangxin Kong, Yin Xian, Yuan Zhang, Xing Xie, Sijun Xie, Aimei Jia, Yixing Ren

**Affiliations:** 1 Department of General Surgery, the Affiliated Hospital of North Sichuan Medical College, Nanchong, 637000 P.R. China; 2 Institute of Hepatobiliary Pancreatic Intestinal Diseases, North Sichuan Medical College, Nanchong, 637000 P.R. China; 3 Chengdu XinHua Hospital Affiliated to North Sichuan Medical College, Chengdu, 610000 P.R. China; 4 Nanchong Psychosomatic Hospital, Nanchong, 637770 P.R. China

**Keywords:** Bariatric surgery, Gout, Hyperuricemia, Uric acid-lowering medication, SUA

## Abstract

**Background/Purpose:**

Patients with gout are at risk for increased serum uric acid (SUA) levels and gout attacks in the short term after undergoing bariatric surgery, and the purpose of this study was to evaluate the benefits of short-term treatment with uric acid-lowering medication after bariatric surgery for the control of gout attacks and SUA levels in patients with gout.

**Methods:**

71 patients who underwent SG from January 2020 to December 2022 were prospectively included. These patients were diagnosed with hyperuricemia before surgery and had a history of gout attacks. Patients were classified into a drug-treatment group (DTG, *n* = 32) and a non-drug-treatment group (NDTG, *n* = 39) according to whether they took uric acid-lowering medication after surgery. Changes in the number of gout attacks, body mass index (BMI), and SUA levels at 1 week, 1 month, 3 months, and 6 months after bariatric surgery were measured in both groups.

**Results:**

In the DTG, 22 patients (68.8%) experienced an increase in SUA within 1 week, 3 patients (9.4%) had an acute attack of gout within the first month, and no patients had a gout attack thereafter. In the NDTG, 35 patients (89.7%) experienced an increase in SUA within 1 week, 7 patients (17.9%) had an acute gout attack within the first month, and 4 patients (10.3%) experienced gout attacks between month 1 and month 3 postoperatively. Both groups were free of gout attacks between the 3rd and 6th postoperative month and showed a significant decrease in SUA and BMI by the sixth month.

**Conclusion:**

In patients with gout, continued use of uric acid-lowering medication after bariatric surgery is beneficial in reducing the number of gout attacks and the risk of rising SUA.

Serum uric acid (SUA) is the final product of purine metabolism and is influenced by factors such as intake and excretion [1–4]. Any condition that increases the production of SUA or reduces its excretion can lead to hyperuricemia [5]. In cases when SUA concentrations exceed saturation levels, urate crystals can form, leading to the development of gout, a condition that can cause severe joint damage and kidney disease [6].

Currently, the mainstream treatment for hyperuricemia focuses on drugs that inhibit uric acid production and promote uric acid excretion, such as allopurinol, febuxostat and topiroxostat (all are xanthine oxidase inhibitors). Probenecid and benzbromarone are also widely used clinically as drugs that inhibit uric acid reabsorption [7]. However, although the effectiveness of these drugs has been demonstrated [8], their clinical use is limited due to serious side effects such as hepatotoxicity, nephrotoxicity and Stevens-Johnson syndrome [9]. Therefore, the exploration of new treatment options has become a necessity.

Obesity and its associated metabolic syndrome are major risk factors for the development of gout, and weight loss has been shown to promote uric acid excretion and improve insulin sensitivity, which has a positive effect against gout attacks [10, 11]. Bariatric surgery is a viable and sustainable weight loss treatment option for population with morbid obesity, and its benefits extend beyond weight loss to include improvements in other metabolic diseases [12].

Related studies have shown that some patients with hyperuricemia experience severe fluctuations in SUA levels and acute gout attacks in the short term following bariatric surgery [13–17], and bariatric surgery has been proven to be an essential factor in triggering acute gout attacks [18]. However, there are limited published reports describing interventions for managing short-term SUA fluctuations and acute gout attacks after bariatric surgery, and the potential benefits of perioperative uric acid-lowering medication in controlling these symptoms have not been clearly established.

## Method

### Study population

We conducted an analysis of 347 patients who underwent laparoscopic sleeve gastrectomy (LSG) at the General Surgery Department of the Affiliated Hospital of North Sichuan Medical College (Nanchong, Sichuan, China) between January 2020 and December 2022. Among them, 71 patients met the inclusion/exclusion criteria and were included in the study. We then divided these patients into two groups based on whether they continued to take urate-lowering drugs after bariatric surgery: drug-treatment group (DTG, *n* = 32) and non-drug-treatment group (NDTG, *n* = 39). All patients were given febuxostat (40 mg/day) preoperatively to control SUA levels, and were treated with non-steroidal anti-inflammatory drugs (e.g., etoricoxib, celecoxib) and colchicine during acute attacks of gout. Patients with gout usually present with severe pain in the joints of the lower limbs, especially the first metatarsophalangeal joints are involved, which is a characteristic feature of gout, and together with imaging tests, such as ultrasound and X-rays, which can assist in the diagnosis of gout if gouty stone formations, joint cavity effusions, and synovitis are observed [19]. For SUA monitoring, we will require patients to fast for more than 8 h before drawing blood, and measure the SUA level through an automatic biochemical analyzer. Hyperuricemia is defined as an SUA level of not less than 360 µmol/L for females or 420 µmol/L for males [20]. All those who were lost to follow-up or not regularly followed up were excluded.

**Fig. 1:**
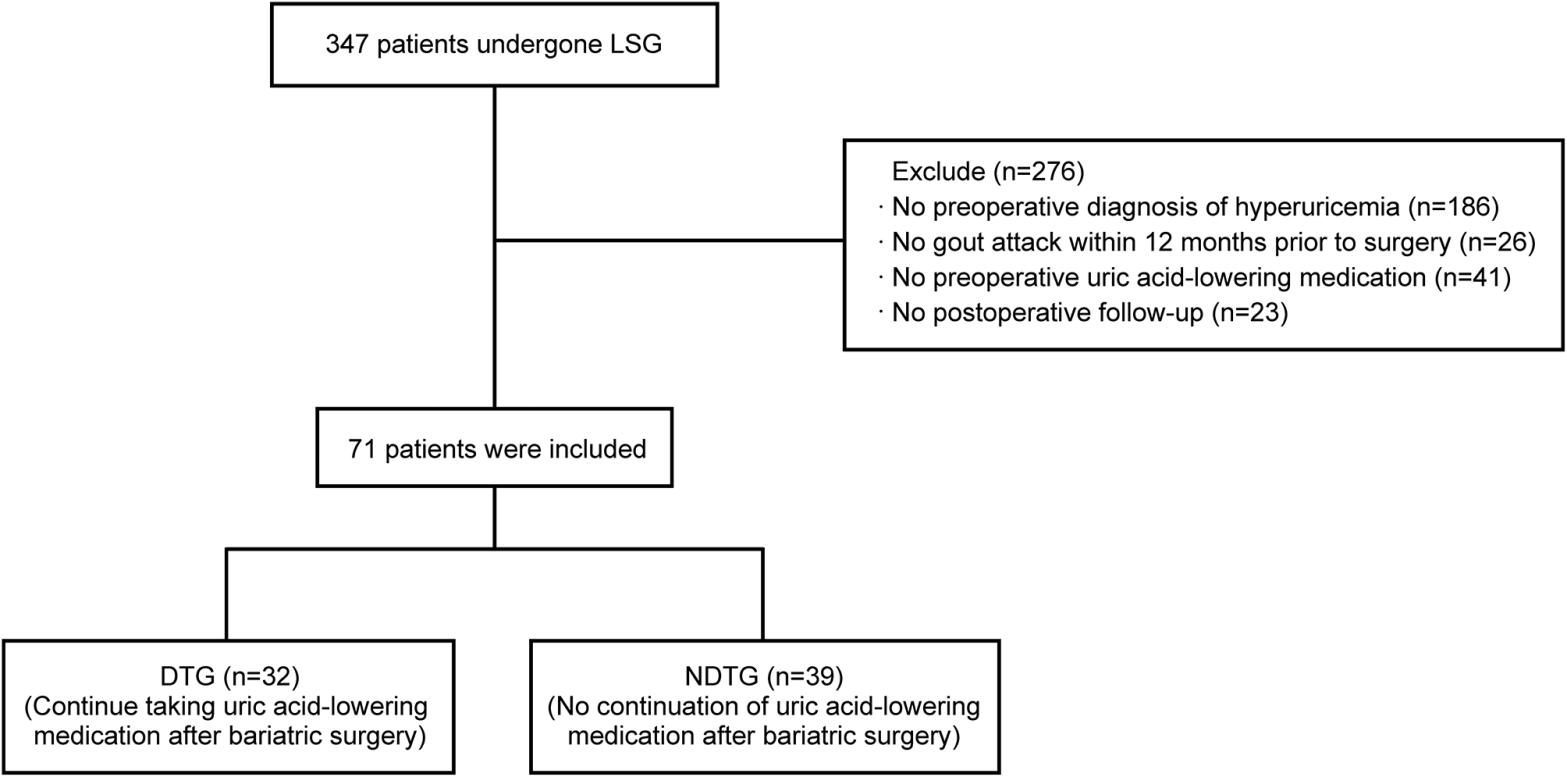
Inclusion/exclusion flow chart

The inclusion criteria and the exclusion criteria are shown in Table 1, and the flow chart is shown in Fig. 1.

The study was approved by the local ethics committee (Ethics Committee of Affiliated Hospital of Sichuan North Medical College) (No. 2023ER189-1), and all patients were informed in detail of the risks of the operation and possible postoperative complications before the operation, and informed consent was signed by the patients or their families (in line with the Declaration of Helsinki).

**Table 1:** The inclusion and exclusion criteria of this study

Inclusion criteria	Exclusion criteria
• Age ranges from 18 to 65 years old	• Previous history of bariatric surgery
• Meet the diagnosis criteria of morbid obesity according to the WHO criteria for obesity in Asian populations [21]	• Allergic to uric acid-lowering drugs or have had previous serious adverse reactions
• Preoperative diagnosis of hyperuricemia	• No postoperative follow-up
• At least 1 gout attack in the 12 months prior to surgery	• Any history of hormone, drug or alcohol use during hospitalization and follow-up
• History of uric acid-lowering medication for at least 6 months prior to surgery	

### Perioperative management

All patients were advised to quit both smoking and drinking, febuxostat was taken until 1 day before surgery. After surgery, a low-purine, high-quality protein liquid diet is given. Diuretic use was minimized, and patients were advised to drink moderate amounts of water, as this will not only reduce the risk of acute postoperative gout attacks but also reduce the incidence of urolithiasis associated with bariatric surgery [22, 23]. The continuation of uric acid-lowering treatment for the patients after surgery depends on the judgement of the same attending doctor at that time. DTG was treated with febuxostat, in the same way as before the operation after excluding relevant contraindications. Both groups were treated with colchicine and nonsteroidal anti-inflammatory drugs (NSAIDS) during acute attacks of gout.

### Follow-up

We conducted follow-up assessments of patients at baseline, 1 week, 1, 3 and 6 months after operation to measure changes in weight, SUA levels, and the frequency of gout attacks. Data for the follow-up period were obtained from medical records of post-discharge follow-up visits to outpatient clinics. Gout attacks were followed up and recorded by one of our clinicians via telephone or face-to-face outpatient consultations. The uric acid-lowering treatment for postoperative patients during the follow-up period is based on the same attending doctor’s comprehensive judgement based on the patient’s renal function indicators monitored at each follow-up visit.

### Statistical analysis

GraphPad Prism software (GraphPad Prism 9) was used for all statistical analyses. Continuous and categorical variables were expressed as mean standard deviation (SD) and number (percentage), respectively. Differences between the two groups were analyzed using the t-test or Mann-Whitney test for continuous data and the chi-square test or Fischer’s exact test for categorical data. Multiple group comparisons were performed by analysis of variance. Post hoc testing of significance between each follow-up and baseline within the same group using the Dunnett test. Logistic regression analyses were used to assess the effect of variables on the number of postoperative gouty attacks. Variables with *P* < 0.1 in univariate analyses were subsequently included in multivariate logistic regression analyses. *P* < 0.05 was considered statistically significant.

## Results

Finally, among 347 patients, 71 patients were included in this study. The majority of patients were female (83.1%, *n* = 59) with a mean age of 32.4 ± 4.7 years, and 16.9% were male with a mean age of 39 ± 5.3 years. The mean BMI was 39.5 ± 5.7 kg/m^2^ for DTG subjects and 38.6 ± 6.6 kg/m^2^ for NDTG subjects. The mean SUA level was 455.3 ± 37.9 µmol/L for the DTG and 472.3 ± 38.3 µmol/L for the NDTG. The mean number of gout attacks in the 12 months prior to bariatric surgery was 1.9 ± 0.6 for DTG and 2.1 ± 0.7 for NDTG (Table 2). There was no statistically significant difference between the two groups (*P* = 0.49).

**Table 2:** Preoperative characteristics of the study population

Parameter	DTG (*n* = 32)	NDTG (*n* = 39)	*P* value
Number of patients Male Female Ages (years)	32 5 27 33.2 (5.6)	39 7 32 33.5 (5.4)_	- - - 0.8767
Weight (kg)	98.5 (12.7)	95.3 (15.8)	0.4923
BMI (kg/m^2^)	39.5 (5.1)	38.6 (6.6)	0.5318
SUA (µmol/L)	455.3 (37.9)	472.3 (38.3)	0.3188
Number of gout attacks	1.9 (0.6)	2.1 (0.7)	0.4898
Creatinine	70.8 (19.4)	78.7 (24.1)	0.1455

Unless specified, data are represented as the mean (SD)

BMI body mass index, SUA serum uric acid, DTG drug treatment group, NDTG non-drug-treatment group

### At 1 week post-surgery

**Fig. 2:**
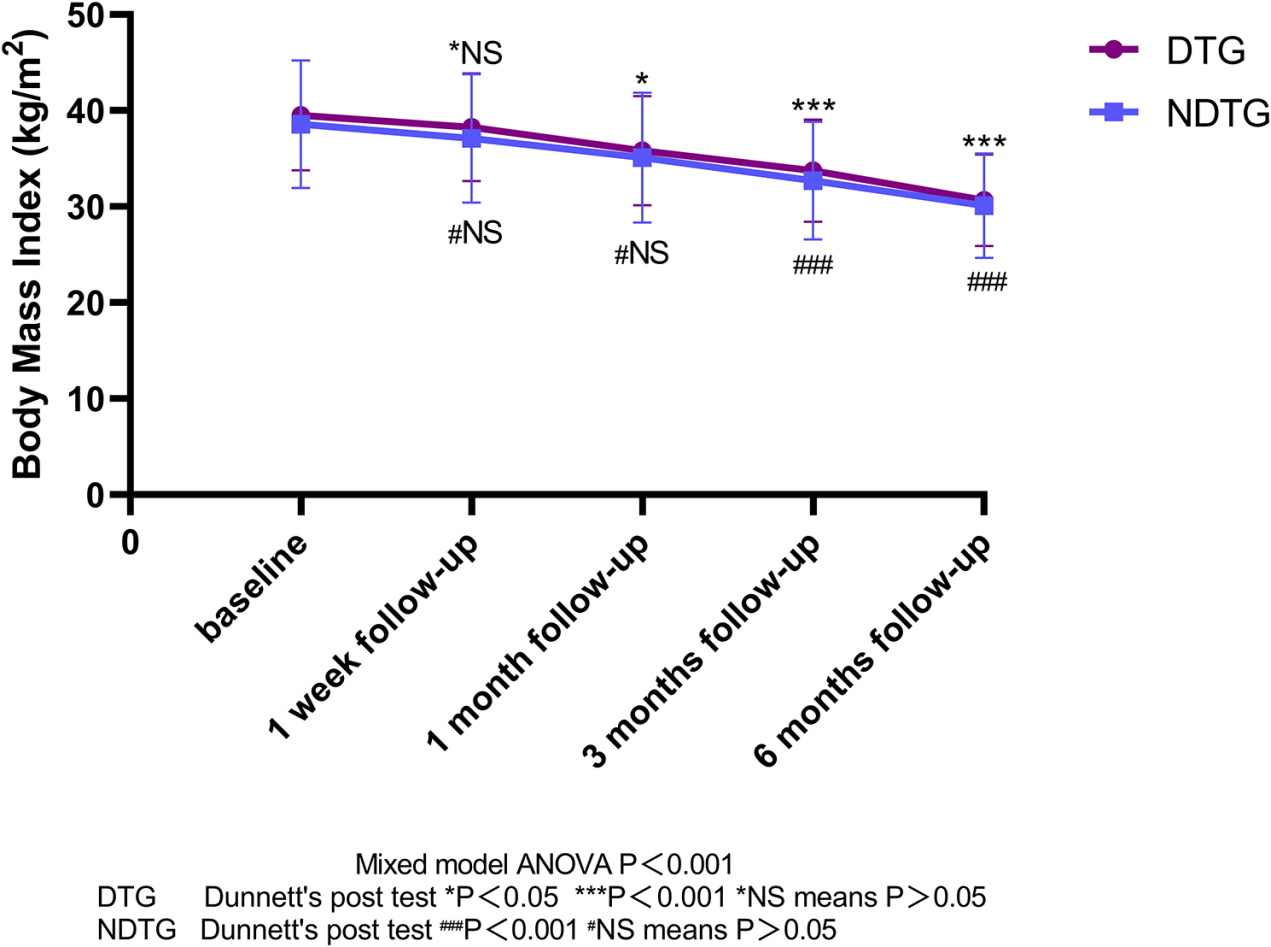
Body mass index at baseline and follow-ups after bariatric surgery

**Fig. 3:**
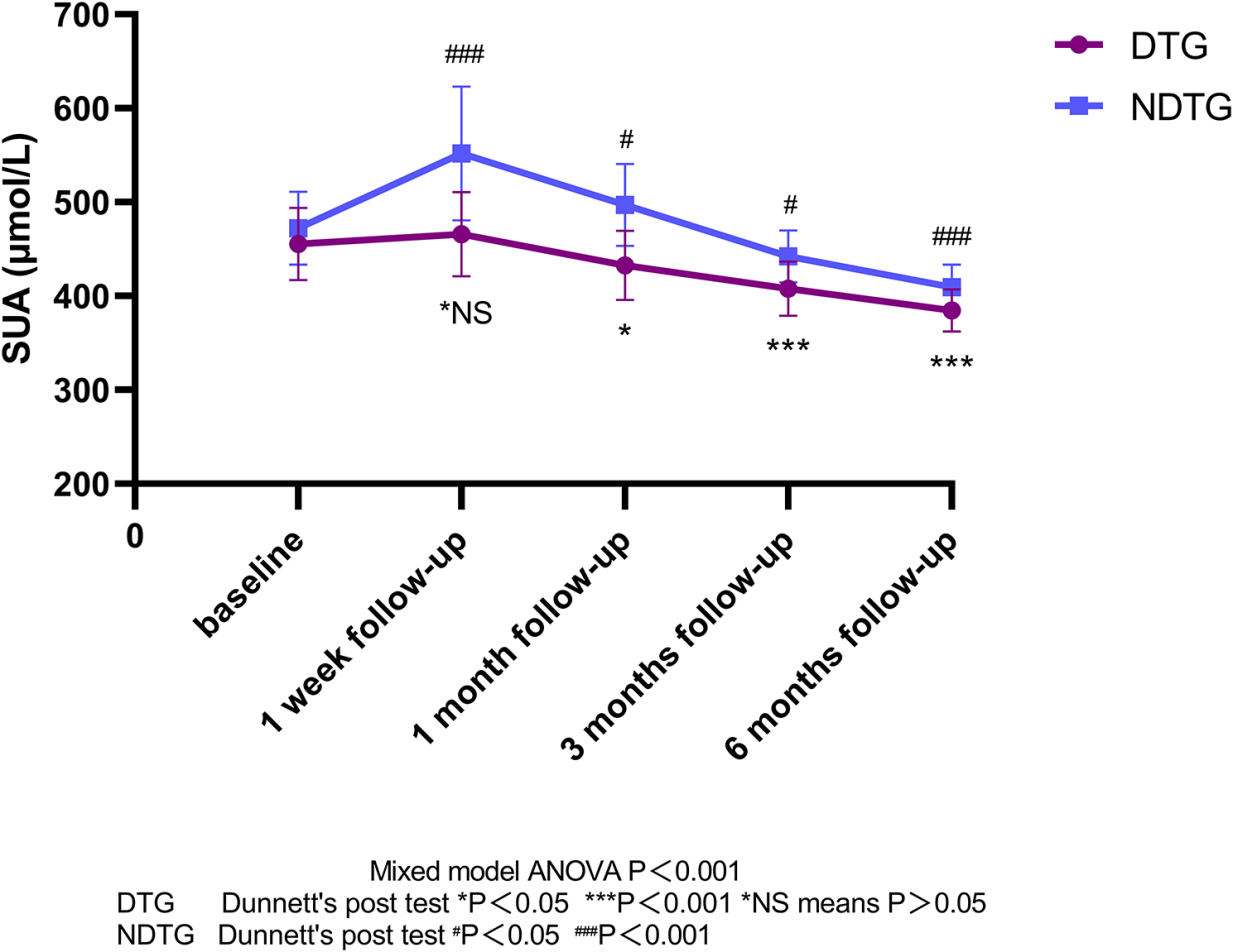
Serum uric acid levels at baseline and follow-ups after bariatric surgery

There was no significant change in BMI in either group compared to baseline (1 week vs. baseline, *P* = 0.3315 for NDTG, *P* = 0.3742 for DTG). Among the DTG, 22 patients (68.8%) experienced an increase in SUA. Overall uric acid level fluctuations were small and nonsignificant (1 week vs. baseline, *P* = 0.3188), and 2 patients (6.2%) had a gout attack. 35 patients (89.7%) in NDTG had a rise in SUA with great fluctuations (*P* < 0.001), and 12.8% of patients (*n* = 5) had at least one acute gout attack within 1 week. All patients in DTG continued to take their medication (Table 3; Figs. 2 and 3).

**Table 3:** Clinical characteristics of all patients included in this study

	All		SUA (µmol/L)		Gout Attack (numbers)		Number of patients continued to take drugs in DTG
	BMI (kg/m^2^)	SUA (µmol/L)	DTG	NDTG	DTG	NDTG	
Baseline	38.9 (6.1)	464.6 (39.0)	455.3 (37.9)	472.3 (38.3)	-	-	32
1 week	37.6 (6.2)	513.1 (73.7)	465.8 (44.1)	551.9 (70.3)	2	5	32
1 month	35.4 (6.2)	463.0 (51.5)	432.6 (36.4)	497.1 (43.2)	1	2	27
3 months	33.2 (5.7)	462.7 (32.6)	407.9 (28.5)	442.2 (27.1)	0	4	12
6 months	30.3 (5.1)	398.1 (26.1)	384.6 (22.2)	409.2 (23.7)	0	0	4
*P* value	<0.05^*αγδ&λΩεσω^	<0.01^*αβδ&ΔλΩεσω^	<0.01^*αγδ&ΔλΩεσω^	<0.001^*αβδ&ΔλΩεσω^	-	-	-

Unless specified, data are represented as the mean (SD)

* *P* < 0.05, α Paired samples t test, β Baseline versus 1 week, γ Baseline versus 1 month, δ Baseline versus 3 months, & Baseline versus 6 months, Δ 1 week versus 1 month, λ 1 week versus 3 months, Ω 1 week versus 6 months, ε 1 month versus 3 months, σ 1month versus 6 months, ω 3months versus 6months

BMI body mass index, SUA serum uric acid, DTG drug treatment group, NDTG non-drug-treatment group

### At 1 month post-surgery

Both groups showed a slight decrease in BMI compared to baseline, and overall SUA levels tended to decrease compared to 1 week postoperatively. In the DTG, SUA levels decreased below baseline, and in the NDTG, SUA levels fell more sharply (*p* < 0.05) compared to 1 week postoperatively and baseline. 1 patient in the DTG group had an acute attack of gout, and 2 patients in the NDTG had 1 acute attack of gout on postoperative days 9 and 22 respectively. 84.4% (*n* = 27) of patients in the DTG continued to take their medication (Table 3; Figs. 2 and 3).

### At 3 months post-surgery

There was some notable reduction in BMI in both groups compared to baseline (*P* < 0.01), with no statistically significant difference in BMI between the two groups (*P* = 0.4503). Moreover, there was a significant decrease in SUA levels in both groups at 3 months compared to 1 week and 1 month postoperatively (*p* < 0.01). No acute attacks of gout occurred in DTG, four times gout attacks occurred in NDTG during month 3 to month 6. Twelve patients in the DTG continued to take uric acid-lowering drugs (Table 3; Figs. 2 and 3).

### At 6 months post-surgery

There was significant decrease in BMI in both groups compared to baseline (*P* < 0.001).Moreover, there was a significant decrease in SUA levels in both groups at 6 months compared to 1 week, 1 month and 3 months postoperatively (*p* < 0.01).No acute attacks of gout occurred in either group. Four patients in the DTG continued to take urate-lowering drugs (Table 3; Figs. 2 and 3).

### Correlation analysis of gouty attacks in postoperative patients

The logistic univariate regression analyses showed values of < 0.1 for preoperative BMI, preoperative SUA, and postoperative continuation of medications (Table 4). Subsequently, these indicators were included in multivariate logistic regression analyses (Table 5), which showed that preoperative BMI and preoperative SUA and were positively associated with postoperative gouty attacks, and postoperative continuation of uric acid-lowering medications was negatively associated with gouty attacks.

**Table 4:** Univariate logistic regression of risk factors associated with postoperative gouty attacks

Variables	OR (95%CI)	*P* value
Sex (male)	1.455 (0.337–6.274)	0.615
Age (years)	1.047 (0.941–1.165)	0.401
Baseline BMI (kg/m^2^)	1.396 (1.170–1.666)	<0.01
Baseline SUA (µmol/L)	1.061 (1.027–1.097)	<0.01
Creatinine (µmol/L)	1.014 (0.989–1.039)	0.273
Continuation of uric acid-lowering drugs	0.263 (0.066–1.045)	0.058

BMI body mass index, SUA serum uric acid

**Table 5:** Multivariate analysis of postoperative gouty attacks by logistics regression

Variables	OR (95%CI)	*P* value
Baseline BMI (kg/m^2^)	1.582 (1.138–2.201)	0.006
Baseline SUA (µmol/L)	1.047 (1.007–1.089)	0.020
Continuation of uric acid-lowering drugs	0.039 (0.002–0.939)	0.046

BMI body mass index, SUA serum uric acid

## Discussion

To the best of our knowledge, this is the first study on the use of uric acid-lowering drugs after bariatric surgery. In this study, patients in both groups achieved better control of SUA levels at 6 months after bariatric surgery, Most DTG subjects experienced a smoother and more gradual decrease in SUA levels over the course of 1 month, avoiding acute attacks of gout due to excessive fluctuations in uric acid. All patients were free of gout attacks during the period of 3–6 months after surgery, and they showed significant decreases in both SUA and BMI at 6 months after surgery. In addition, we found through logistic regression analysis that preoperative BMI and SUA levels were also positively correlated with postoperative gout attacks.

Obesity and metabolic syndrome have been prospectively studied as clinically relevant risk factors for hyperuricemia and gout [24], and patients with severe obesity tend to have a higher risk of developing hyperuricemia [25–28]. As a chronic disease, patients diagnosed with hyperuricemia or gout may require long-term or even lifelong medication, with medication compliance and side effects being major issues. Therefore, the use of uric acid-lowering drugs for hyperuricemia remains controversial [29]. Furthermore, traditional drug therapy may have limitations for patients with obesity and gout, as treatment with uric acid-lowering drugs alone is often less effective and may have certain drawbacks [30, 31].

Current research has demonstrated the effectiveness of weight loss achieved through dietary intervention or weight loss surgery in lowering SUA levels and preventing gout attacks [11, 15, 30–32]. Several pieces of research evidence have suggested that bariatric surgery can decrease the long-term incidence of hyperuricemia and gout, and even enable certain patients to discontinue urate-lowering therapy [15, 33]. Moreover, besides weight loss, the reduction in SUA may be linked to a reduction in the systemic inflammatory response associated with the metabolic syndrome (This response is triggered by changes in adipokines and pro-inflammatory cytokines resulting from bariatric surgery). Additionally, it may also be associated with the reduction in insulin resistance [34], which can be combined with other reported benefits of bariatric surgery [35, 36]. Nevertheless, the recurrence of postoperative hyperuricemia and the repeated reports of acute gout attacks should not be ignored.

There is documented evidence that high preoperative SUA levels and suspension of urate-lowering therapy are important risk factors for acute gout attacks [37, 38]. Our study discovered that bariatric surgery followed by uric acid-lowering medication in patients with a preoperative diagnosis of hyperuricemia and a history of acute gout attacks resulted in reduced short-term fluctuations in SUA levels, thereby lowering the number of gout attacks in the short term. Moreover, we also found that bariatric surgery was effective in reducing the morbidity of hyperuricemia, as this will be demonstrated in our subsequent long-term follow-up.

One potential cause of postoperative SUA level fluctuations in patients with gout that we cannot ignore is the perioperative diet. According to the Chinese Expert Consensus on Precision Obesity Metabolic Surgery (2022 Edition) [39], our center recommends a specific diet plan for patients following surgery. Patients are asked to consume a liquid diet for 2 weeks after surgery, gradually transitioning to a dregs-free, full liquid diet that is easy to digest. From 3 to 4 weeks after surgery, patients are asked to consume a semi-liquid diet with easily digestible foods. From 1 to 2 months after surgery, patients are recommended to consume a soft diet that includes a variety of high-quality protein sources such as meat, fish and protein supplements. However, this type of diet may increase uric acid levels by enhancing purine metabolism, and may lead to gout attacks [40]. The catabolic state induced by perioperative caloric restriction has also been suggested as a potential cause of gout attacks [31]. The American Society for Metabolic and Bariatric Surgery (ASMBS) and the International Federation for Surgery of Obesity and Metabolic Diseases (IFSO) advocate a high-protein, low-carbohydrate, and low-fat postoperative diet [41, 42], however, low-carbohydrate diets induce mobilization of fatty acids from abdominal and hepatic stores, which, together with increased protein intake, are associated with increased purine release and concomitant ketogenic state, formation of β-hydroxybutyrate and acetoacetate, impairing renal excretion of uric acid and consequently increasing the number of acute gout attacks [43]. In contrast to this dietary pattern, the traditional Chinese diet practices place more emphasis on grain and vegetable intake, with a lower protein content. However, soup consumption may lead to micronutrient deficiencies after surgery [44, 45]. Therefore, in addition to the standard dietary recommendations, we also advise our patients to control their protein intake and receive appropriate rehydration therapy. Beyond that, in our center, patients without previous hyperuricemia also experience short-term fluctuations in SUA after bariatric surgery, for whom it may be clinically relevant to investigate the impact of perioperative diet on short-term fluctuations in SUA.

Our study had several limitations that need to be acknowledged. First, since this study is an observational study, coupled with strict inclusion/exclusion criteria (Table 1), resulting in a small sample size, this may limit the generalizability of our findings. Second, although the diagnosis of gout in most patients relies on imaging examinations, because this is an observational study and the particularity of bariatric surgery patients, there are still a small number of patients whose gout diagnosis relies on symptoms and chief complaints. In the future, during the study, we will collect synovial fluid specimens and imaging evidence from all patients to support the diagnosis. Third, it is acknowledged that various bariatric surgery procedures can yield diverse effects on patients’ absorption [46, 47]. However, this study specifically focused on laparoscopic sleeve gastrectomy, with no inclusion of patients who underwent Roux-en-Y gastric bypass (RYGB) or other types of surgeries. Therefore, the potential impacts of other weight loss surgery methods on drug absorption were not considered. Finally, the follow-up period was relatively short, and more than 80% of the patients included in this study were women, since gout is more common in men, this may limit the possibility of generalizing our findings. To address these limitations, further prospective studies with larger sample sizes and longer follow-up periods, including multicenter collaborations, are needed to better assess the effects of medication on SUA control and gout attack prevention in patients with gout undergoing bariatric surgery.

## Conclusion

In summary, our study provides evidence for uric acid-lowering therapy after bariatric surgery in patients with gout. Our findings demonstrate a significant increase in SUA levels after surgery and subsequent acute attacks of gout, highlighting the importance of uric acid-lowering therapy after surgery. However, the decision to discontinue therapy should be based on the individual patient’s condition. Despite some limitations of our study, our results provide valuable insights into the management of hyperuricemia and gout in bariatric surgery patients, and further studies are needed to confirm and extend our findings.

## Data Availability

The datasets used and/or analysed during the current study available from the corresponding author on reasonable request.
